# Evolution and Quantitative Comparison of Genome-Wide Protein Domain Distributions

**DOI:** 10.3390/genes2040912

**Published:** 2011-11-09

**Authors:** Arli A. Parikesit, Peter F. Stadler, Sonja J. Prohaska

**Affiliations:** 1 Computational EvoDevo Group, Department of Computer Science, University of Leipzig, Härtelstraße 16–18, D-04107 Leipzig, Germany; E-Mail: arli@bioinf.uni-leipzig.de; 2 Interdisciplinary Center for Bioinformatics, University of Leipzig, Härtelstraße 16–18, D-04107 Leipzig, Germany; E-Mail: studla@bioinf.uni-leipzig.de; 3 Bioinformatics Group, Department of Computer Science, University of Leipzig, Härtelstraße 16–18, D-04107 Leipzig, Germany; 4 Max Planck Institute for Mathematics in the Sciences, Inselstrasse 22, D-04103 Leipzig, Germany; 5 Fraunhofer Institut für Zelltherapie und Immunologie—IZI Perlickstraße 1, D-04103 Leipzig, Germany; 6 Department of Theoretical Chemistry, University of Vienna, Währingerstraße 17, A-1090 Wien, Austria; 7 Center for non-coding RNA in Technology and Health, University of Copenhagen, Grønnegårdsvej 3, DK-1870 Frederiksberg C, Denmark; 8 Santa Fe Institute, 1399 Hyde Park Rd., Santa Fe, NM 87501, USA

**Keywords:** protein domains, HMM models, GO classification, functional genome annotation, Eukarya

## Abstract

The metabolic and regulatory capabilities of an organism are implicit in its protein content. This is often hard to estimate, however, due to ascertainment biases inherent in the available genome annotations. Its complement of recognizable functional protein domains and their combinations convey essentially the same information and at the same time are much more readily accessible, although protein domain models trained for one phylogenetic group frequently fail on distantly related sequences. Pooling related domain models based on their GO-annotation in combination with *de novo* gene prediction methods provides estimates that seem to be less affected by phylogenetic biases. We show here for 18 diverse representatives from all eukaryotic kingdoms that a pooled analysis of the tendencies for co-occurrence or avoidance of protein domains is indeed feasible. This type of analysis can reveal general large-scale patterns in the domain co-occurrence and helps to identify lineage-specific variations in the evolution of protein domains. Somewhat surprisingly, we do not find strong ubiquitous patterns governing the evolutionary behavior of specific functional classes. Instead, there are strong variations between the major groups of Eukaryotes, pointing at systematic differences in their evolutionary constraints.

## 1. Introduction

The protein repertoire of an organism provides summary information on its metabolic and regulatory capabilities. It should be possible at least in principle to identify large-scale trends in evolution such as the increased complexity of transcriptional regulation, chromatin-related mechanisms, or post-transcriptional silencing, by comparing the proteomes among species. This approach is indeed widely used. The comparison of predicted protein contents among related species is, for instance, an integral part of most genome papers.

The identification of gene families and the determination of orthologs, or at least homologs, is an extremely difficult and time-consuming task in comparisons across kingdoms or even across the three domains of life. The obstacles are not only of a technical nature. Proteins are composed of recognizable protein domains that can be recombined in a combinatorial fashion to produce new functionalities over large time-scales. Individual proteins often have multiple ancestors that contributed with different domains to an extant protein [1,2]. From a biochemical point of view, furthermore, many protein domains can be associated with particular molecular interactions with which they contribute to the protein's overall function. From an evolutionary perspective, furthermore, they form quite well-defined and stable units of selection. As an alternative to reconstructing protein evolution, one thus may focus on tracing the distribution of individual domains [3–6]. In a recent study of chromatin evolution, we demonstrated that it is indeed feasible to determine large-scale trends in regulatory capabilities based on domain content [7].

The distribution of domains within proteins is not completely uniform. For instance, about fifty specific domains are preferentially found in alternatively spliced exons and hence systematically lacking in some protein variants [8]. As most proteins contain more than a single domain, domain combinations are of particular interest when aiming at a more detailed understanding of the novel functions [6]. Interestingly, domains differ in their intrinsic propensity to co-occur with many different other domains. This versatility, however, is primarily dependent upon the position of the domain at the end of proteins and their occurrence in single domain proteins. This can be explained by fusions and fissions as the most frequent genomic operations creating novel domain combinations [9]. This is an ongoing evolutionary process. On the other hand, some “promiscuous” domains, in particular those involved in protein-protein interactions, have a propensity to appear in particularly wide variety of different domain architectures [10]. For instance, there are many animal-specific or even vertebrate-specific domain-combinations [11].

More global trends can be uncovered by considering aggregate statistics of domains and domain combinations. The average number of domains in a protein, for instance, increases systematically along the human lineage [6]. Network analysis of domain co-occurrences, furthermore, demonstrates a growing core of combinations in multicellular organisms [12].

Typically, studies of this type are based on existing protein annotations derived primarily from genomic sequence data. Popular data sources are, e.g., the protein annotation compiled in KEGG or ENSEMBL. Protein domains from Pfam [13] domains were used in [11]. The studies [6,7] are based on the SUPERFAMILY database [14], whose HMM models in turn are based on the SCOP (Structural Classification of Proteins) domain definitions [15]. Both the protein annotation and the collections of domains, however, suffer from substantial biases [16]:

(1) Our knowledge of protein domains is by far not complete, although most protein domains in well-studied model organisms are evolutionarily very old, while innovation of protein domains at the same time is a relatively infrequent phenomenon [17,18]. The majority of “plant-specific” DNA binding domains, for instance, originated much earlier then the comparably recent expansion into the diverse gene families present in higher plants [19]. Unrecognized domains thus have to be attributed in many cases to insufficient sensitivity of the domain annotation procedure. Non-globular segments of proteins, in particular transmembrane regions and signal peptides, furthermore have a hydrophobic bias leading to problematic domain models and subsequently to completely wrong function assignments inherited from these domain models [20].
(1) Domains are typically annotated on protein sequences stored in sequence databases. These “protein models” in turn are the result of computational procedures that combine the genomic DNA sequence, EST and cDNA data, and homology-based predictions. Differences in the amount of available experimental evidence can lead to dramatic ascertainment biases [16]: The number of annotated domains in SUPERFAMILY 1.73, for example varies by more than a factor of four within eutherian mammals (64,225 domains in human *versus* 14,748 in the alpaca) although one would expect these species to have a very similar gene complement.

The first point can be addressed by pooling related domain models derived from data for different phylogenetic groups, albeit at the expense of losing resolution regarding structural and functional differences among domains belonging to the same family or superfamily. There does not seem to be an easy remedy for the ascertainment biases when currently available databases are used. In [16] we therefore proposed to bypass existing genome annotations and instead to estimate domain occurrence data by combining *de novo* gene prediction with HMM-based domain annotation of the predicted protein structures. We found that the number of domains found by this procedure correlates very well with the annotation compiled in the SUPERFAMILY database for both human and yeast. Furthermore, consistent estimates are obtained for closely related species such as the apes. This implies that cross-species comparisons are more meaningful when using a consistent *de novo* annotation pipeline than based on currently available protein databases. We note that false positives of the gene prediction step are not much of a problem for our purposes since the predicted amino acid sequences do not match valid protein domain models. False negatives, on the other hand, affect our results. Hence, we trade completeness for a relatively unbiased annotation so that estimates of domain content are consistent between different genomes. Taken together, this allows quantitative comparisons of domain-occurrences and co-occurrences at least at a statistical level.

**Figure 1:**
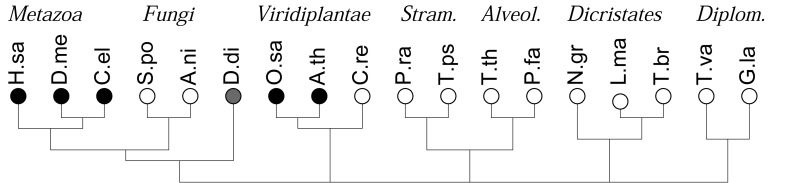
Phylogenetic distribution of the species considered in this work following [21], showing the disputed deepest nodes unresolved. Abbreviations and genome assembly: H.sa: *Homo sapiens* (hg19); D.me: *Drosophila melanogaster* (BDGP5.13); C.el: *Caenorhabditis elegans* (WS200); S.po: *Schizosaccharomyces pombe* (EF1); A.ni: *Aspergillus niger* (CADRE); D.di: *Dictyostelium discoideum* (DDB); O.sa: *Oryza sativa* (OSV6.1); A.th: *Arabidopsis thaliana* (TAIR9.55); C.re: *Chlamydomonas reinhardtii* (Chlre4); P.ra: *Phytophthora ramorum* (Phyra1_1); T.ps: *Thalassiosira pseudonana* (Thaps3); T.th: *Tetrahymena thermophila* (tta1_oct2008); P.fa: *Plasmodium falciparum* (PlasmoDB-7.0); N.gr: *Naegleria gruberi* (Naegr1); L.ma: *Leishmania major* (Lmj_20070731_V5.2); T.br: *Trypanosoma brucei* (Tb927_May08_v4); T.va: *Trichomonas vaginalis* (TrichDB-1.2); G.la: *Giardia lamblia* (WBC6); *Stram.:* Stramenopiles; *Alveol.:* Alveolata; *Diplom*.: Diplomonada. Multicellular species are marked by a black dot, unicellular ones with a white dot. The gray dot marks the slime mold.

As a first application of this approach, we recently investigated the co-occurrences of four major types of DNA binding domains (zinc fingers, leucine-zipper, HMG-box domains, and winged-helix domains) and observed a strong and statistically highly significant anti-correlation of the four different domains. In contrast, evolutionarily related DNA binding domains readily co-occur in DNA binding proteins [16]. In many genomes, in particular in the rather compact genomes of simple unicellular eukaryotes, however, the total number of genes and domains that can be annotated is too small for a meaningful statistical evaluation. Here we show that this limitation can be overcome by pooling domains in terms of domain families or even at the level of functional classes of domains. The gain in statistical power, however, is paid for by a loss of resolution and the additional effort required for the aggregation of domain models in meaningful groups. The focus of study is different from previous approaches, which concentrated primarily on the patterns and dynamics of domain evolution in individual protein families, see e.g., [1], or consider the genomic repertoire of protein domains, e.g., [3,6,18]. Here we are interested in global trends of domain co-occurrence at genome- and kingdom-wide scales, Figure 1.

## 2. Results and Discussion

### 2.1. Results

**Figure 2:**
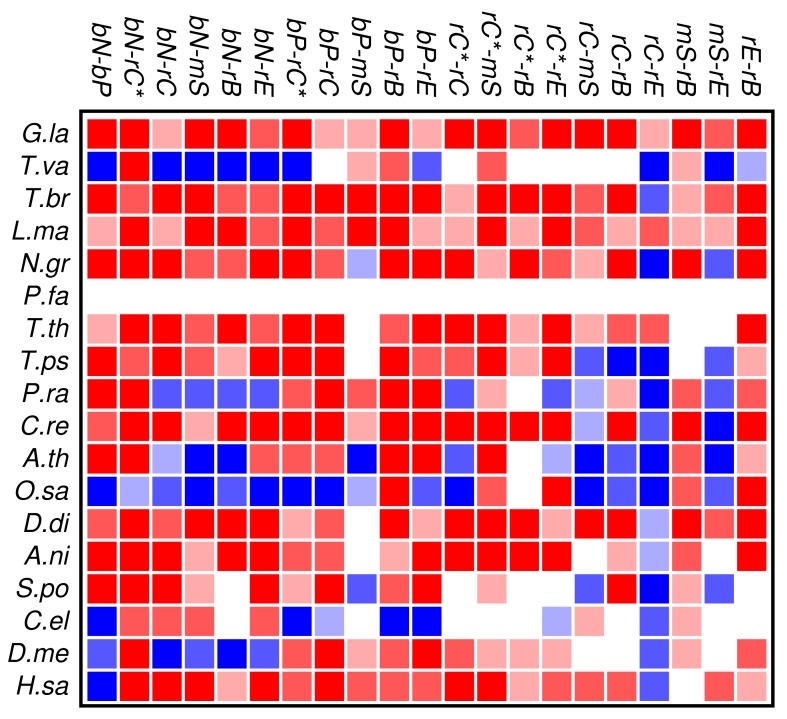
Summary of domain co-occurrences in 18 eukaryotic genomes. Colors indicate the statistical significance of co-occurrence *n*(*C, D*) ≫ *E*(*C|D*) (red) and of avoidance *n*(*C, D*) ≪ E(*C|D*) (blue). Significance levels on individual comparisons are shown in three levels of color saturation for *p* < 0.001, 0.001 ≤ *p* < 0.01, and 0.01 ≤ *p* < 0.1, respectively. See section 3.2 for the abbreviations of the function classes.

The results of the co-occurrence analysis at the level of GO classes is summarized in Figure 2 for the complete set of domains. For the GO classes compiled in section 3.2, we observe some interesting global patterns. With the exception of the functional classes rE (regulation of enzymatic activity) and rC (regulation of chromatin in a narrow sense) there is no pattern of conserved avoidance. In fact, most other combinations of domain functions are at least weakly positively correlated.

With respect to the phylogenetic distribution of co-occurrence patterns, the most interesting observation is a trend towards wide-spread avoidance in particular in multicellular plants, and—to a lesser extent—also in animals. Among unicellular species, only *Trichomonas* and *Phytophthora* show similar patterns of functional avoidance. The lack of significant signals is at least in part explained by the small number of proteins that can be annotated by *ab initio* methods.

**Figure 3:**
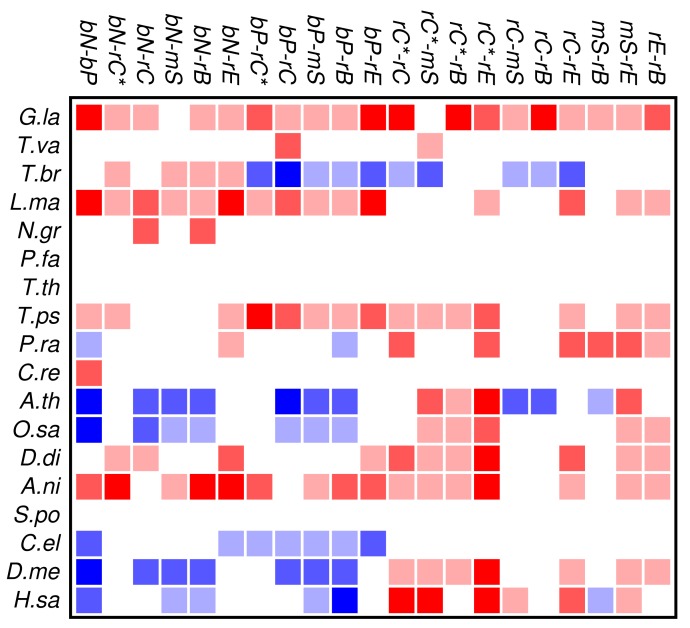
Summary of domain co-occurrences of functional classes of protein domains in zinc finger proteins. See Figure 2 for the color scheme.

Zinc finger proteins are one of the largest single classes of proteins [31]. In Figure 3 we investigate to what extent the occurrence and co-occurrence of other domains is influenced by the additional presence of a zinc finger domain. Surprisingly, we find that patterns of positive or negative correlation among domain functions are enhanced within zinc finger proteins. In fact, we find much more significant deviations from the expectation even though the sample size is of course much smaller than in Figure 2. In particular, we again observe that domain avoidance is most common within multicellular organisms, where they affect in particular the two groups of nucleic acid and protein binding domains. We suspect that this statistical pattern derives from recent rapid expansions of particular protein families. An example would be the mammalian-specific KRAB-ZNF protein comprising hundreds of closely related transcription factors [32].

Several organisms, in particular *Tetrahymena* and *Plasmodium*, have only few zinc finger genes, so that a global statistical analysis of this protein family cannot provide meaningful results. At present we do not have a good explanation for the wide-spread avoidance among other domain functions in the many zinc finger genes of *Trypanosoma*.

**Figure 4:**
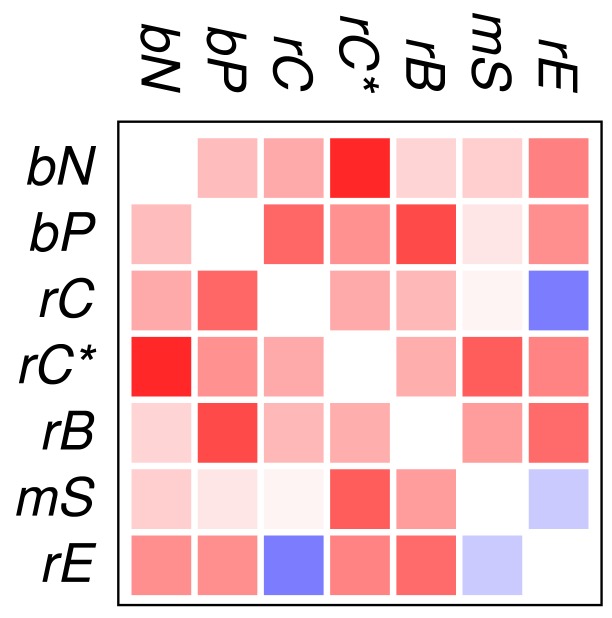
Summary of co-occurrence data. See text for details.

Figure 2 and Figure 3 show that the mutual relationships of a few many, but certainly not all, GO classes are observed coherently across the major groups of Eukarya. Due to the large differences in genome size and domain numbers it makes little sense to compute a summary statistic by adding up the counts of occurrences across species: such data would be dominated by the large, gene-rich multicellular organisms. Instead we employ a simple voting procedure, associating scores of +3, +1, −1, and −3 only with the two most significant levels of co-occurrence and avoidance, respectively. Figure 4 displays these scores averaged over the 18 species for the all proteins. An analogous plot for zinc finger proteins does not reveal global patterns because there are much fewer and weaker significant signals (not shown).

We find that most of the domain GO-classes are at least weakly positively correlated, in part reflecting the fact that the protein domains can have promiscuous functions, in part possibly also because the domains investigated here are mostly involved in binding and regulatory processes. Surprisingly, the only combination that shows strong avoidance across all data sets is *regulation of chromatin vs.regulation of catalytic activity* (rC:rE). This effect is not visible in comparison to the set rC* of domains associated with chromatin-regulation. The latter in particular contains also enzymatic domains such as kinases and phosphatases involved in chemical modifications of histones [7].

In contrast to the data set comprising all domain pairs, we observe much less coherence among the domain classes in zinc finger proteins. On the other hand, we observe that the clade-specific patterns become more pronounced in the zinc finger data set. This indicates that the evolutionary trends within this group of proteins is dominated by lineage-specific influences rather than global correlations of domain functions.

To our surprise, we did not observe a systemic anti-correlation of the domains involved in saccharide metabolism (mS) and regulation of enzymatic activity (rE), resp., with the binding and chromatin associated domains. For the mS group, correlations with functional classes are weak, while rE co-occurs readily with binding domains but avoids the core set of chromatin associated domains (rC). In retrospect, the positive correlation of rE and bP makes sense as regulators of enzymatic activity have reason to bind to enzymes. This also explains the co-occurrence with the rC* set, which contains in particular also histone modifying enzymes. We have at present no good explanation, however, why we also observe co-occurrence with nucleic acid binding.

### 2.2. Discussion

Protein domains become the natural level of description of protein evolution in particular when very large evolutionary time-scales are of interest. Broad cross-species comparisons are dependent upon unbiased estimates of the number and genomic distribution of protein domains. Thus ascertainment biases that can arise from large differences in the coverage of gene annotation and from the use of very specific domain models need to be avoided or at least reduced as much as possible. Here we have investigated, therefore, to what extent it is feasible to compare patterns of functional protein classes across all major groups of Eukarya based on automatic *de novo* gene annotation and pooling of domain-models into larger functional classes.

In both respects, substantial improvements should be feasible for future, more comprehensive studies: in particular, it appears promising to combine transcript-based gene annotation with trained, instead of general-purpose, *ab initio* gene prediction. We expect that such an extension will increase the accuracy of domain estimates in particular in genomes with unusual structure such as ciliates or kinetoplastids. The incomplete and potentially biased set of gene models available already in early stages of genome annotation projects can be expected to provide sufficient training data for our purposes. A reduction of the phylogenetic bias of domain models, on the other hand, will also require the development of a solid theoretical framework to inter- and extrapolate protein domain models well beyond the phylogenetic range in which the domain was annotated and hence was available for constructing the HMM.

## 3. Experimental Section

### 3.1. Genome-Wide Domain Annotation

We consider the 18 species with sequenced genomes shown in Figure 1, covering the entire phylogenetic range of the eukaryotes. Gene predictions were performed using genscan [22,23]. Following [16], we split long chromosomes into overlapping fragments of about 500 kb to accommodate genscan's restriction on input length. Protein sequences were extracted directly from the genscan predictions. Duplicate predictions in the overlaps between fragments were removed. A summary is given in Table 1. Although this procedure in general yields good results, as shown previously for mammals and yeast [16], care must be taken in case of unusual genome structures. In the case of polycistronic mRNAs, as in the case of the kinetoplastids (*Leishmania* and *Trypanosoma*) we may expect a tendency to overcount co-occurrences since polycistrons are not correctly split into individual functional units. Short scaffolds, as in the case of the *Tetrahymena* data, on the other hand, lead to underestimates. The extreme A + T content of *Plasmodium*, furthermore may account for the relative small number of predicted genes and the low number of reliably annotated domains [24,25].

In order to obtain comparable domain predictions across the widely different eukaryotic genomes we took all Hidden Markov Models (HMMs) [26–28] provided by the SUPERFAMILY database [14,29]. We used HMMER 3.0rc1 [30] to map the HMMs to amino acid sequences predicted by genscan with the cut-off *E* ≤ 10^−3^. Only the best scoring domain from a set of overlapping domains is considered further. The result is, for each predicted protein, a list of non-overlapping domains. Here we use zinc fingers as an illustrative example since they form one of the most abundant classes of DNA-binding domain; other wide-spread domain families can be analyzed in the same manner. Operationally, we classify a genscan prediction as “zinc finger gene” if it contains at least one C2H2 domain (SCOP family 57668).

**Table 1:** Summary of gene and domain annotation.

**Species**	**all genes**		**zinc finger genes**	

	**genes**	**domains**	**genes**	**domains**
H.sa	118,894	139,016	5,370	9,096
D.me	28,889	62,906	1,005	2,452
C.el	12,432	8,752	158	310
S.po	3,578	8,146	37	68
A.ni	8,112	24,334	82	250
D.di	5,323	24,496	27	99
O.sa	64,109	108,972	369	745
A.th	20,135	49,974	192	686
C.re	13,268	41,576	29	79
P.ra	16,701	53,410	107	299
T.ps	8,766	22,006	35	71
T.th	2,011	3,028	3	2
P.fa	1,439	3,466	6	14
N.gr	10,748	28,016	17	50
L.ma	4,560	20,554	25	121
T.br	5,143	20,710	286	1,641
T.va	19,251	49,214	25	63
G.la	11,251	42,324	35	116

### 3.2. GO Annotation

Version 1.75 of the SUPERFAMILY database offers a “Structural Domain Functional Ontology” providing functional and phenotypic annotations of protein domains at the ***superfamily*** and ***family*** levels [29]. Since any protein can be annotated by multiple functions, it is clear that membership in GO annotation classes does lead to a partition of the set of protein domains into functional groups. In this work we use the following seven groups:

bN *binding of nucleic acids*: GO:0003676 at superfamily level.
bP *binding of proteins* with potential nuclear localization: GO:0005515 superfamily level.
rC *regulation of chromatin* GO:0016568 at superfamily level.
rC* *regulation of chromatin* as determined in [7], comprising a combination of family and superfamily level.
rB *regulation of binding*: GO:0051098 at superfamily level.
rE *regulators of enzymatic activity*: GO:0050790 at superfamily level.
mS *metabolism of saccharides*: GO:0005976 at superfamily level.

The five functional groups bN, bP, rC, rC* and rB were chosen because of their expected preferential co-occurrence with zinc finger genes. Both bN and bP play an important role for gene regulation by transcription factors and are among the most abundant GO classes. The choice of the two variants of chromatin-associated domains rC and rC* is motivated by our previous work on the co-occurrence of protein domains that can act as readers, writers, and erasers of histone modification [7], which revealed changes in the co-occurrence patterns within this group. The domain groups rE and mS were intended as negative controls as we did not expect them to correlate in a particular way with either nucleic acid or protein binding domains (bN, bP).

We then annotated the membership of a domain in the functional groups. Table 2 shows that only few domains are associated with more than one group. The result is, for each predicted protein, a list of non-overlapping domains and their group memberships. When estimating the co-occurrence of two GO-classes *C* and *D* we correct for the fact that a domain *x* can be a member of both *C* and *D* by counting these cases with a weight of 1/2.

**Table 2:** Overlaps between the 7 functional groups defined in the text.

	bN	bP	rC	rC*	mS	rB	rE
bN	112	4	4	4	0	8	6
bP		118	6	7	0	4	21
rC			25	11	0	1	0
rC*				27	0	1	2
mS					14	0	0
rB						15	1
rE							55

### 3.3. Co-Occurrence Analysis

For each of the 18 species, we separately evaluated the number of domain co-occurrences and the number of genes in which two domains *x* and *y* co-occur. Let *n_x_* be the total number of annotated domains belonging to group *x*. The simplest estimate for the expected number of domain co-occurrences is *E*(*x, y*) = *n_x_n_y_/n_g_*, where *n_g_* is the number genes in the genome under consideration. This estimate does not account for biases arising from the non-uniform distribution of domains over genes. Let *n_d_*(*i*) be the number of domains predicted for protein *i*, and let *n_d_* *=* Σ*_i_* *n_d_*(*i*) be the total number of domains. Then the number of domains of group *x* that occur in genes that also contain a *y* domain can be estimated by

(1) $$E(x|y)=(n_{x}/n_{d})\sum_{i:y\in i}\left(n_{d}(i)-1\right)$$

where the sum runs over all genes that contain a domain belonging to group *y*. These expectations are then compared with the number of empirically observed co-occurrences *n*(*x, y*). We speak of *co-occurrence* of domain families or groups if *n*(*x, y*) ≫ *E*(*x|y*)*, E*(*y|x), E*(*x, y*) and of *avoidance* if *n*(*x, y*) ≪ *E*(*x|y*)*, E*(*y|x*)*, E*(*x, y*). The statistical significance of an observed difference between *n*(*x, y*) and *E*(*x|y*)*, E*(*y|x*)*,* or *E*(*x, y*), respectively, is determined under the assumption that *n*(*x, y*) is drawn from a Poisson distribution.

## 4. Conclusions

Despite obvious shortcoming of the gene finding procedure in organisms with unusual genome structure or extreme sequence composition and the unavoidable limitations of the domain annotation, some global patterns nevertheless become visible in this pilot study. The classes of protein domains investigated here are all involved in binding and/or regulation. There does not seem to be an intrinsic tendency of these domains to segregate into different proteins or protein families. In the multi-cellular organisms with large genomes and large gene families, however, we observe a strong signal of avoidance between several functional groups of protein domains, Figure 2. This may be a result of the expansion and diversification of large families of paralogous genes and their use for specific tasks in the regulation of cellular processes. Furthermore, we observe substantial differences in the domain co-occurrence patterns of distant lineages, emphasizing the importance of lineage-specific histories and constraints.
